# Lipopolysaccharide Causes Acquired CFTR Dysfunction in Murine Nasal Airways

**DOI:** 10.1002/ohn.1143

**Published:** 2025-01-26

**Authors:** Jessica W. Grayson, T. Graham Norwood, Shaoyan Zhang, Daniel Skinner, Do‐Yeon Cho, Bradford A. Woodworth

**Affiliations:** ^1^ Department of Otolaryngology–Head and Neck Surgery University of Alabama at Birmingham Heersink School of Medicine Birmingham Alabama USA; ^2^ Departments of Surgery and Veterans Affairs Birmingham Division of Otolaryngology Birmingham Alabama USA; ^3^ Gregory Fleming James Cystic Fibrosis Research Center University of Alabama Birmingham Birmingham Alabama USA

**Keywords:** CFTR, chronic rhinosinusitis, chronic sinusitis, histopathology, lipopolysaccharide, nasal potential difference, sinusitis, transepithelial ion transport

## Abstract

**Objective:**

Cystic fibrosis (CF) is a clinical entity defined by aberrant chloride (Cl^−^) ion transport causing downstream effects on mucociliary clearance (MCC) in sinonasal epithelia. Inducible deficiencies in transepithelial Cl^−^ transport via CF transmembrane conductance regulator (CFTR) has been theorized to be a driving process in recalcitrant chronic rhinosinusitis (CRS) in patients without CF. We have previously identified that brief exposures to bacterial lipopolysaccharide (LPS) in mammalian cells induces an acquired dysfunction of CFTR in vitro and in vivo. The objective of the current study is to evaluate whether LPS generates a model of acquired CFTR dysfunction murine nasal airways.

**Study Design:**

Basic science.

**Setting:**

Laboratory.

**Methods:**

CFTR^+/+^ murine nasal airways were irrigated with 2 µg/mL LPS or control vehicle twice daily for 1 week and transepithelial Cl^−^ transport assessed with the nasal potential difference (NPD) assay. Histopathologic evaluation included the number of lymphoid aggregates, as well as the epithelial and subepithelial heights.

**Results:**

Transepithelial Cl^−^ secretion by NPD was markedly reduced in mice exposed to LPS (in mV, −0.14 ± 7.7 vs control, −6.98 ± 7.15, *P* < .05), while amiloride‐sensitive voltage was preserved (6.38 ± 5.09 vs control, 7.36 ± 2.87, *P* = .99). Histopathology demonstrated significantly higher lymphoid aggregates per high‐power field (2.3 ± 0.9 vs 1.1 ± 0.7, control, *P* < .01) and increased epithelial height (in µm, 40.88 ± 13.9 vs control, 25.32 ± 6.26, *P* < .05).

**Conclusion:**

Twice daily irrigation with LPS in murine nasal airways over 1 week led to acquired defects in transepithelial Cl^−^ transport. This animal model provides an excellent means to test the contributions of acquired CFTR dysfunction to CRS and test CFTR correctors and potentiators that might improve MCC.

## Introduction

Cystic fibrosis (CF) is an autosomal recessive disorder characterized by the deficiency or dysfunction of the CF transmembrane conductance regulator (CFTR). The normal physiological functions of respiratory epithelium are closely tied to transepithelial ion and water transport pathways.^1^, ^2^ CFTR serves as the primary apical anion channel in the sinonasal epithelium and is essential for maintaining the proper hydration and ionic balance of airway surface liquid. When CFTR function is inhibited, it reduces the clearance of respiratory pathogens by decreasing ciliary beat frequency (CBF), airway surface liquid thickness and hydration, and mucociliary clearance (MCC).^3^, ^4^, ^5^, ^6^, ^7^ Consequently, CFTR deficiency increases the risk of airway bacterial infections, leading to chronic inflammatory sinus disease—a frequent cause of morbidity in the CF population.^8^, ^9^, ^10^, ^11^, ^12^, ^13^, ^14^, ^15^, ^16^

Genetic mutations in individuals with CF contribute to the characteristic phenotypic expression of the disease. However, the processing, endocytic recycling, and function of wild‐type CFTR can also be significantly impaired by many external factors, including high altitude/hypoxemia, hypoxia, inflammation, cigarette smoke exposure, and bacterial exoproducts.^17^, ^18^, ^19^, ^20^, ^21^, ^22^, ^23^ Given that non‐CF CRS shares phenotypic similarities and involves similar gram‐negative bacteria as CF CRS, it is plausible to consider acquired dysfunction in CFTR as a causative etiology in this population.^24^, ^25^ For example, Prince et al identified *Pseudomonas* in approximately 24% and gram‐negative bacteria in general in 30% of consecutive CRS patients undergoing sinus surgery with endoscopically directed cultures.^26^ Additionally, silent carriers of CFTR mutations are more susceptible to CRS, representing a spectrum of CFTR‐related disorders.^27^ MCC is known to be reduced in non‐CF CRS but can be re‐established once infection and inflammation in the sinuses are resolved.^28^, ^29^

Lipopolysaccharide (LPS) is a highly pro‐inflammatory molecule found on the outer membrane of gram‐negative bacteria, such as *Pseudomonas aeruginosa*, a common pathogen in CRS.^30^, ^31^, ^32^, ^33^, ^34^ LPS triggers the swift and dynamic translocation of the nuclear transcription factor NF‐kappa B, which controls the expression of numerous genes.^35^, ^36^, ^37^ Infections of the sinuses with gram‐negative bacteria are often particularly severe and resistant to treatment.^38^, ^39^, ^40^, ^41^, ^42^, ^43^ Our previously published research has shown that brief 4‐hour exposures to LPS can induce partial CFTR dysfunction, independent of inflammation. This dysfunction is linked to the creation of reactive oxygen species, which carbonylate CFTR and reduce its open probability in patch‐clamp studies.^44^, ^45^ However, it remains unclear whether these effects persist with repeated, intermittent exposures in an in vivo nasal model.

The objective of the present study was to create a reliable, reproducible in vivo murine nasal epithelial model of acquired CFTR dysfunction.

## Materials and Methods

This study was approved by the Institutional Animal Care and Use Committee (IACUC) at the University of Alabama at Birmingham Heersink School of Medicine.

### In Vivo Nasal Potential Difference (NPD) Measurements

All chemicals were purchased from Sigma‐Aldrich (St. Louis, Mo.). *P. aeruginosa* LPS dissolved in phosphate buffered saline (PBS) at a concentration of 2 µg/mL or vehicle control solution was instilled intranasally (1 mL twice a day by syringe) in C57/BL6 CFTR^+/+^ male mice for a period of 7 days (n = 10 per condition). These mice have normal CFTR function at baseline. Our previous short‐term exposures at 4 hours with 10 µg/mL embedded in a thermal‐based gel induced an acquired CFTR dysfunction from oxidative damage to the channels rather than from inflammation. For the purpose of the current study, we used a 5‐fold reduction in concentration without the thermal‐based gel to ensure short exposures and sufficient recovery of CFTR dysfunction mediated by oxidative damage. The mice were anesthetized at the end of 7‐day exposure after a 24‐hour washout period and NPD assays performed, as described previously.^46^, ^47^, ^48^, ^49^, ^50^ The initial step in NPD measurement protocol requires the perfusion of the nasal cavities of anesthetized mice (C57BL/6) with Ringer's solution (pH 7.3) containing 140 mM NaCl, 5 mM KCl, 1 mM MgCl_2_, 2 mM CaCl_2_, 10 mM HEPES, and 100 µM amiloride to inhibit Na^+^ absorptive pathways. Next, a low Cl^−^‐containing solution was perfused (NMDG [N‐methyl‐D‐glucamine], 6 mM Cl^−^, pH 7.3) followed by the activation of CFTR‐mediated Cl^−^ secretion with forskolin 20 µM in the perfusate. Because of the continuous presence of amiloride (50 µM) and the complete replacement of Na^+^ with a membrane‐impermeant cation (140 mM NMDG in the perfusion solution), hyperpolarization reflects Cl^−^ secretion rather than cation absorption. All traces were interpreted in blinded fashion.

### Histopathology

Following NPD measurements, the mice underwent euthanization and the nasal cavities assessed for histologic inflammation. After fixation in 10% formalin, tissue blocks were decalcified, grossly sectioned, embedded in paraffin, thinly sectioned, mounted on glass slides, and stained with hematoxylin and eosin for analysis under the light microscopy by a head and neck pathologist (WCB) blinded to experimental group. Two sections from the anterior and posterior portions of the sinonasal cavity of each murine snout were evaluated for gross evidence of inflammatory infiltrate, the number of lymphoid aggregates per high power field, and epithelial and subepithelial thickness (considered a surrogate marker for airway inflammation) by 2 blinded judges.^51^, ^52^

### Statistical Analysis

Statistical evaluation was completed with GraphPad prism version 10.0.2. Continuous variables were assessed using unpaired Student's *t‐*test. For the NPD assay, Sidak's multiple comparisons post hoc analysis was performed. A *P* value of <.05 was considered significant.

## Results

### CFTR‐Mediated Anion Transport Is Markedly Reduced in a Murine Model of LPS‐Induced Acute Inflammation

**Figure 1:**
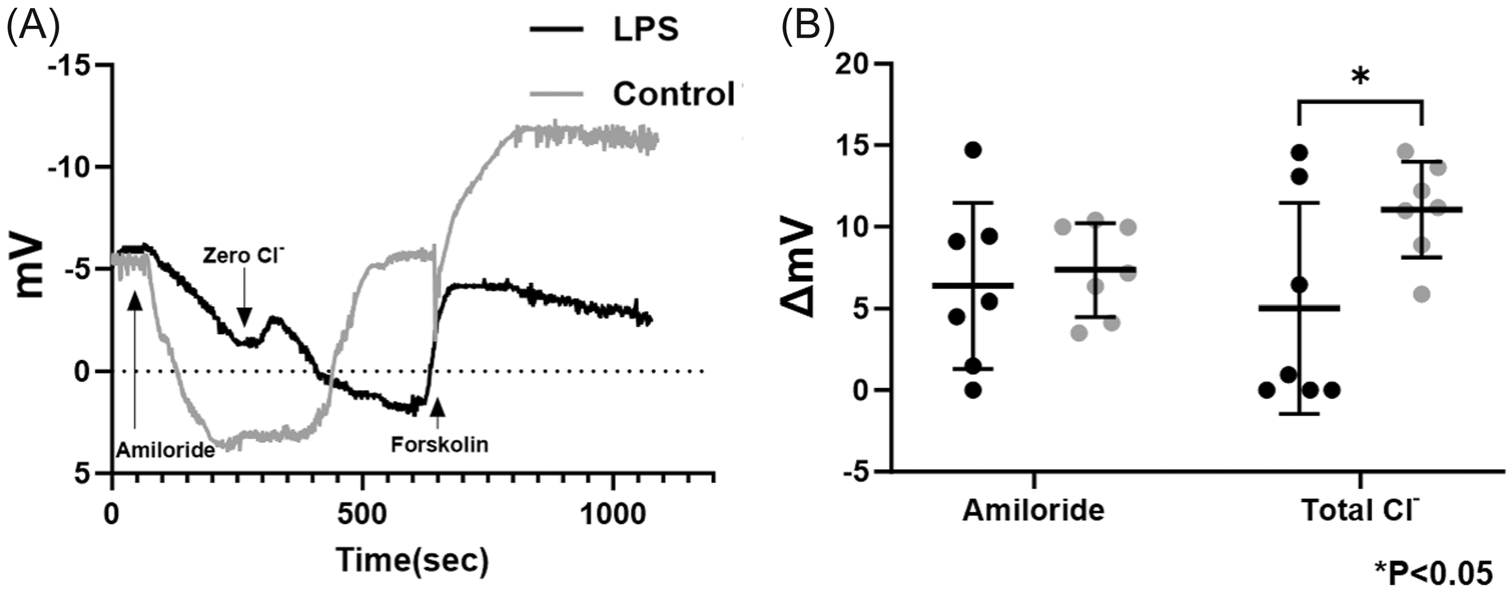
Murine nasal potential difference (NPD) measurements demonstrate decreased transepithelial Cl^−^ transport in vivo in response to lipopolysaccharide (LPS)‐induced acute inflammation (A). Mice underwent a standardized NPD protocol. Depicted are representative tracings of LPS‐exposed mice and PBS controls. (B) Summary data demonstrate that amiloride‐sensitive voltage was similar, but total Cl^−^ transepithelial transport was significantly diminished in LPS‐treated mice (*P* < .05).

Murine NPD assays were performed following twice daily LPS (2 µg/mL) or PBS control irrigations for 1 week. Seven mice in each cohort has usable tracings. Figure 1A shows typical NPD recordings from LPS‐exposed mice and controls. Summary data that illustrate the results from both groups are shown in Figure 1B. Amiloride‐sensitive voltage (in mV) did not significantly differ between groups (LPS, 6.38 ± 5.09 vs control, 7.36 ± 2.87, n = 7 per cohort, *P* = .99). Total Cl^−^ transport, including passive egress through endogenously activated CFTR and other pathways (low Cl^−^) and maximal activation of CFTR with forskolin, was significantly diminished in the LPS‐exposed mice (5.09 ± 6.46 vs 11.05 ± 2.95, *P* < .05).

### LP‐Induced Acute Inflammation In Vivo

**Figure 2:**
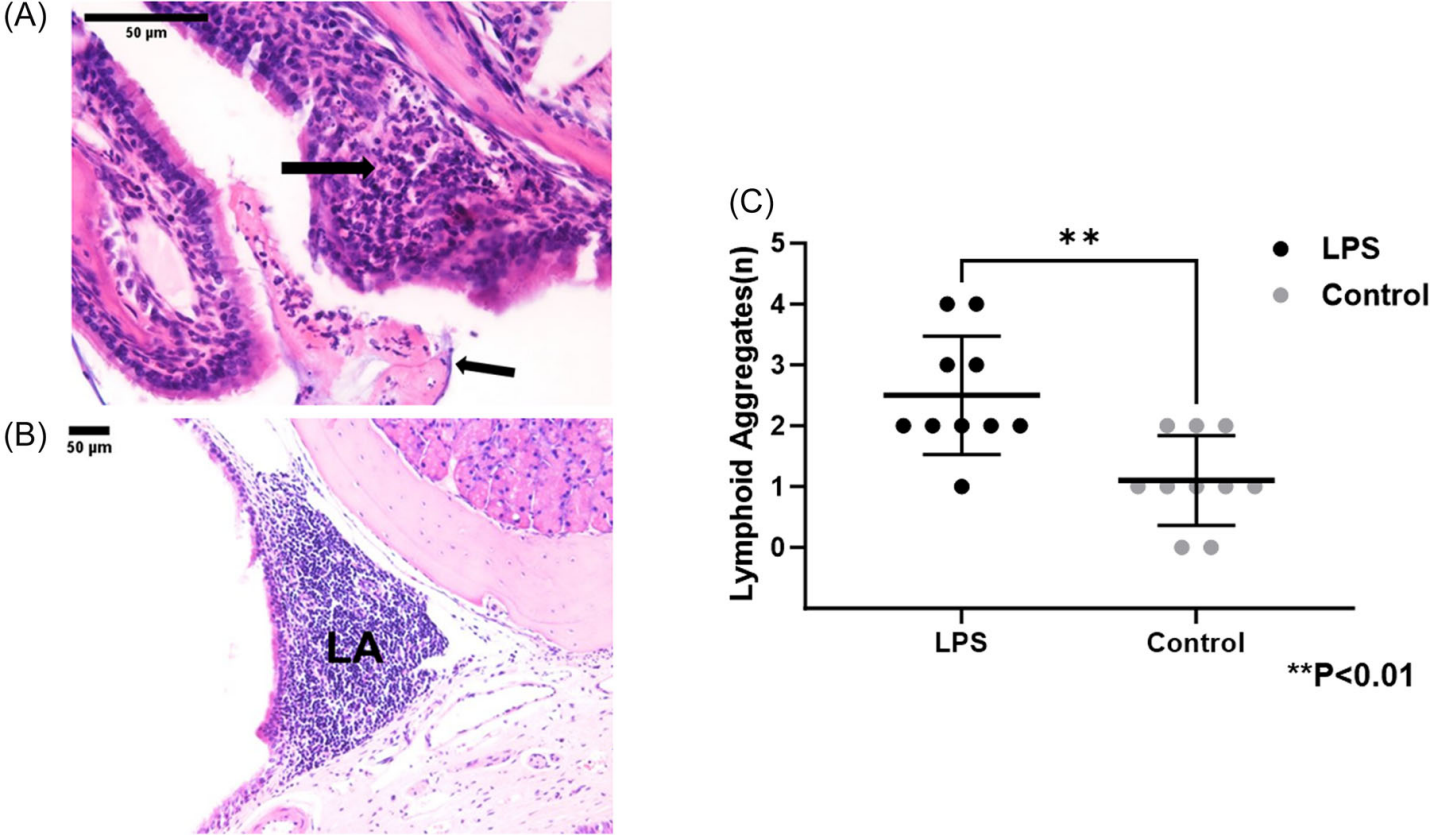
(A, B) Histologic sections of murine snouts revealed LPS‐induced inflammation in the form of subepithelial neutrophils (left, large arrow), exudate (left, small arrow), and lymphoid aggregates (LA, right). (C) Lymphoid aggregates were present in significantly greater numbers in LPS‐exposed mice (*P* < .01).

**Figure 3:**
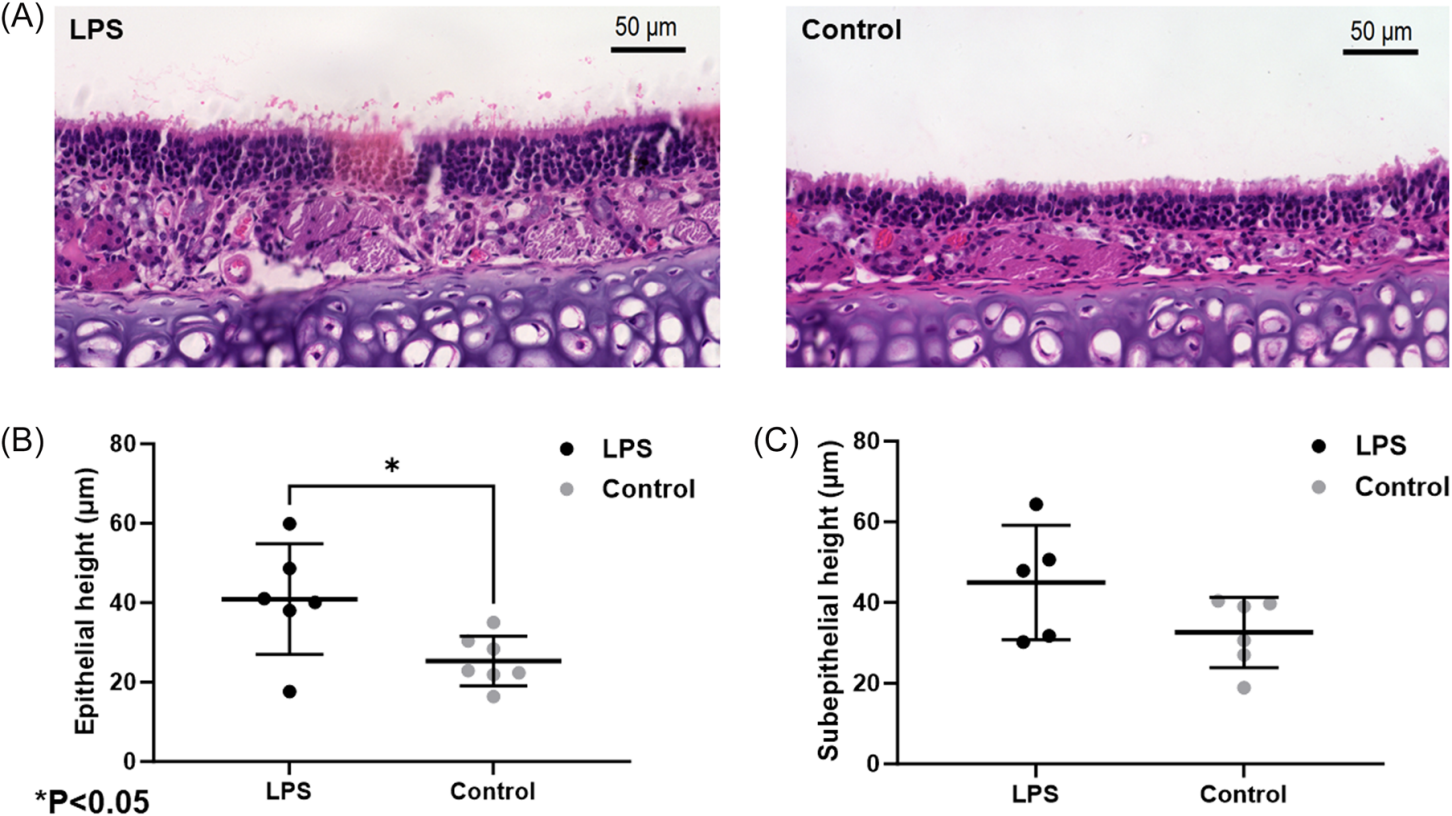
Comparison of epithelial and subepithelial height in (A) histologic sections of LPS‐treated and control murine nasal epithelium. Summary data regarding (B) epithelial and (C) subepithelial height. There was a significant increase in epithelial height in LPS‐treated mice (*P* < .05).

Epithelial neutrophilic infiltration, surface exudate, and lymphoid aggregates were prominent in nasal airways from LPS‐exposed mice (Figure 2A and B). Lymphoid aggregates were present in significantly higher numbers in the LPS mice (2.5 ± 0.9 vs 1.1 ± 0.7, control, *P* < .01) (Figure 2C). Histology was also assessed for changes in epithelial and subepithelial height in LPS and control mice (Figure 3A). Epithelial height was increased in the murine nasal airways (in µm; LPS, 40.88 ± 13.9 vs control, 25.32 ± 6.26, *P* < .05) (Figure 3B). The subepithelial height was not significantly increased in LPS mice but was greater than the control cohort (44.98 ± 14.22 vs control, 32.61 ± 18.68, *P* = .10) (Figure 3C).

## Discussion

This study demonstrates a significant reduction in CFTR‐mediated anion transport in a murine model subjected to LPS‐induced acute inflammation. The NPD assays performed on mice exposed to LPS twice daily for a week revealed marked differences in ion transport compared to control mice treated with PBS. Specifically, while amiloride‐sensitive I_SC_ did not show significant differences between the LPS‐exposed group and controls, total Cl^−^ transport was notably reduced in the LPS group. This reduction includes both passive chloride egress through endogenously activated CFTR and maximal CFTR activation by forskolin, indicating that chronic, sub‐maximal exposures to LPS impair CFTR function in vivo.

Histological analyses confirmed the presence of acute inflammation in the LPS‐exposed mice. Subepithelial neutrophilic infiltration was significantly more pronounced in the nasal airways of LPS‐treated mice compared to controls, indicating a robust inflammatory response. Additionally, the LPS group exhibited a higher number of lymphoid aggregates per high‐power field, suggesting marked immune activation in these mice. Increased epithelial thickness, often considered a marker of epithelial inflammation, was also observed in the nasal airways of LPS‐treated mice, further corroborating the inflammatory effects induced by LPS.

These findings suggest, but do not prove, that the induction of LPS‐induced inflammation affects CFTR function in the nasal airways. Our previously published findings identified a mechanistic sequence where (1) LPS exposure induces reactive oxygen species, (2) reactive oxygen species cause oxidative damage to CFTR, and (3) CFTR function is decreased through diminished open probability of the channels.^45^ This effect would be persistent in vivo where there is constant exposure to LPS in gram‐negative infections until the bacteria are cleared. The current study administered LPS by irrigant every 12 hours, which represents multiple acute, but intermittent, exposures over 1 week. The nasal mucosa had a long duration of recovery between exposures. Our previous data noted recovery of CFTR function after 3 hours when the exposure was removed.^45^ Thus, we postulate in the current study that the induction of inflammation with exposure and alteration of the epithelium is the source of the findings.

Regardless of mechanism, the diminished CFTR‐mediated anion transport in LPS‐exposed mice suggests that the presence of LPS in CRS patient sinuses may exacerbate recalcitrance to treatment by further disruption of MCC. The significant inflammatory response observed, characterized by neutrophilic infiltration and increased lymphoid aggregates, underscores the potential for LPS‐induced inflammation to disrupt epithelial integrity and contribute to the pathophysiology of gram‐negative CRS. Other factors, such as hypoxia,^22^, ^53^, ^54^ can cause CFTR dysfunction in any form of CRS where there are hypoxic sinuses and low oxygen tension in the sinus mucosa. Clearly, gram‐negative bacteria have an additional microbial advantage that can cause persistent mucociliary dysfunction. Further research is needed to establish whether the induction of inflammation is the cause of CFTR dysfunction in the model, as well as the potential therapeutic strategies to mitigate these LPS‐induced alterations in CFTR function and airway inflammation.^55^, ^56^, ^57^, ^58^, ^59^, ^60^

## Conclusion

The present study suggests that exposure to LPS alters active transepithelial ion transport in murine nasal epithelium and leads to acquired defects in CFTR‐mediated anion secretion. Our results indicate that in sinonasal epithelia, LPS may confer significant defects in CFTR activity, elicit persistent mucociliary dysfunction in CRS, and form a localized CF‐like phenotype in vivo. These findings have important implications concerning the role of pro‐inflammatory bacterial components in the pathogenesis of CRS and demonstrate the experimental utility of a murine model for characterizing CFTR function following exposure to various sinonasal insults.

## Author Contributions

**Jessica W. Grayson**, acquired and interpreted the data, revised the article for important intellectual content, approved the version to be published, took public responsibility for appropriate portions of the content; **T. Graham Norwood**, acquired and interpreted the data, revised the article for important intellectual content, approved the version to be published, took public responsibility for appropriate portions of the content; **Shaoyan Zhang**, acquired and interpreted the data, revised the article for important intellectual content, approved the version to be published, took public responsibility for appropriate portions of the content; **Daniel Skinner**, acquired and interpreted the data, revised the article for important intellectual content, approved the version to be published, took public responsibility for appropriate portions of the content; **Do‐Yeon Cho**, acquired and interpreted the data, revised the article for important intellectual content, approved the version to be published, took public responsibility for appropriate portions of the content; **Bradford A. Woodworth**, conceived and designed the work, interpreted the data, drafted the article, approved the version to be published, took public responsibility for appropriate portions of the content.

## Disclosures

### Competing interests

The authors certify that they have no affiliations with or involvement in any organization or entity with any financial interest in the subject matter or materials discussed in this manuscript.

### Funding source

This work was supported by the National Institutes of Health (NIH)/National Heart, Lung, and Blood Institute (R01 HL133006‐05), National Center for Complementary and Integrative Health (R21AT01223‐01), and the Cystic Fibrosis Foundation Research Grant (002481G221) to B. A. W., as well as NIH/National Institutes of Allergy and Infectious disease (K08AI146220 and R21AI168894‐01) to D. Y. C.
